# Evidence of Climate-Induced Range Contractions in Bull Trout *Salvelinus confluentus* in a Rocky Mountain Watershed, U.S.A

**DOI:** 10.1371/journal.pone.0098812

**Published:** 2014-06-04

**Authors:** Lisa A. Eby, Olga Helmy, Lisa M. Holsinger, Michael K. Young

**Affiliations:** 1 University of Montana, Wildlife Biology Program and Department of Ecosystem and Conservation Sciences, College of Forestry and Conservation, Missoula, Montana, United States of America; 2 U.S. Department of Agriculture Forest Service, Rocky Mountain Research Station, Fire Sciences Laboratory, Missoula, Montana, United States of America; 3 U.S. Department of Agriculture Forest Service, Rocky Mountain Research Station, Forestry Sciences Laboratory, Missoula, Montana, United States of America; James Cook University, Australia

## Abstract

Many freshwater fish species are considered vulnerable to stream temperature warming associated with climate change because they are ectothermic, yet there are surprisingly few studies documenting changes in distributions. Streams and rivers in the U.S. Rocky Mountains have been warming for several decades. At the same time these systems have been experiencing an increase in the severity and frequency of wildfires, which often results in habitat changes including increased water temperatures. We resampled 74 sites across a Rocky Mountain watershed 17 to 20 years after initial samples to determine whether there were trends in bull trout occurrence associated with temperature, wildfire, or other habitat variables. We found that site abandonment probabilities (0.36) were significantly higher than colonization probabilities (0.13), which indicated a reduction in the number of occupied sites. Site abandonment probabilities were greater at low elevations with warm temperatures. Other covariates, such as the presence of wildfire, nonnative brook trout, proximity to areas with many adults, and various stream habitat descriptors, were not associated with changes in probability of occupancy. Higher abandonment probabilities at low elevation for bull trout provide initial evidence validating the predictions made by bioclimatic models that bull trout populations will retreat to higher, cooler thermal refuges as water temperatures increase. The geographic breadth of these declines across the region is unknown but the approach of revisiting historical sites using an occupancy framework provides a useful template for additional assessments.

## Introduction

Freshwater ecosystems host a disproportionately large amount of the Earth's biodiversity, including many fish species of economic and cultural value [1], yet account for an outsize share of globally imperiled species [2], [3]. Aquatic organisms in freshwater ecosystems are expected to be particularly sensitive to climate shifts because most are ectothermic and have a relatively narrow thermal range for growth and survival [4], [5]. Bioclimatic models accounting for climate change predict an array of phenological changes and range shifts in freshwater aquatic species [6], [7]. Alteration in the timing of life history events has been relatively widely observed [8], [9]. In contrast, confirmation of predictions that stenothermic cold-water fishes should be undergoing distributional shifts to cooler, high-elevation refuges has been elusive [10], particularly in North America [11].

The northern Rocky Mountains, U.S.A. is undergoing climate-mediated shifts e.g., reduced annual snowpack, earlier annual peak snowmelt, and winter precipitation switching from snow to rain, that are contributing to changes in hydrologic and thermal regimes [12], [13], [14]. Summer water temperatures have increased up to 0.3°C/decade [15] and summer base flows are declining [16]. The most stenothermic coldwater fish in the northern Rocky Mountains is the bull trout (*Salvelinus confluentus*) [17], which is listed as threatened under the U.S. Endangered Species Act. Juvenile bull trout rear in cold stream reaches across the upper elevations of river networks, with the upstream extent of individual populations limited by channel size and gradient [18]. Regional temperature increases associated with climate change have led to dire predictions about the persistence of this species in the U.S. [17], [19], but there is little empirical evidence of climate-related shifts. An additional complexity is attributing changes in occupancy directly to climate change [20]. Wildfire is a frequent natural disturbance that can lead to the decades-long elevation of summer stream temperatures because of the loss of shade from riparian vegetation [21], [22]. A recent increase in fire severity and size in the western U.S. has been linked to climate change [23]. Similarly, climate projections favor headwater invasions by less thermally restricted nonnative species such as brook trout (*S. fontinalis*) [24] that can reduce bull trout occupancy [25].

In this study, we repeated a late 20th-century inventory of bull trout occupancy within a river network that encompasses a broad temperature and elevation gradient. Our objective was to compare site-scale abandonment and colonization probabilities to determine whether they differed and if they were associated with landscape features such as wildfire occurrence and severity, habitat attributes including gradient, width, large wood, temperature, and elevation, and biotic variables such as proximity to strongholds of migratory adults and brook trout presence. If the range of bull trout contracted in response to climate change, we expected site-level abandonment probabilities to be greatest at the warmest sites and to exceed those for colonization.

## Materials and Methods

### Study area

**Figure 1:**
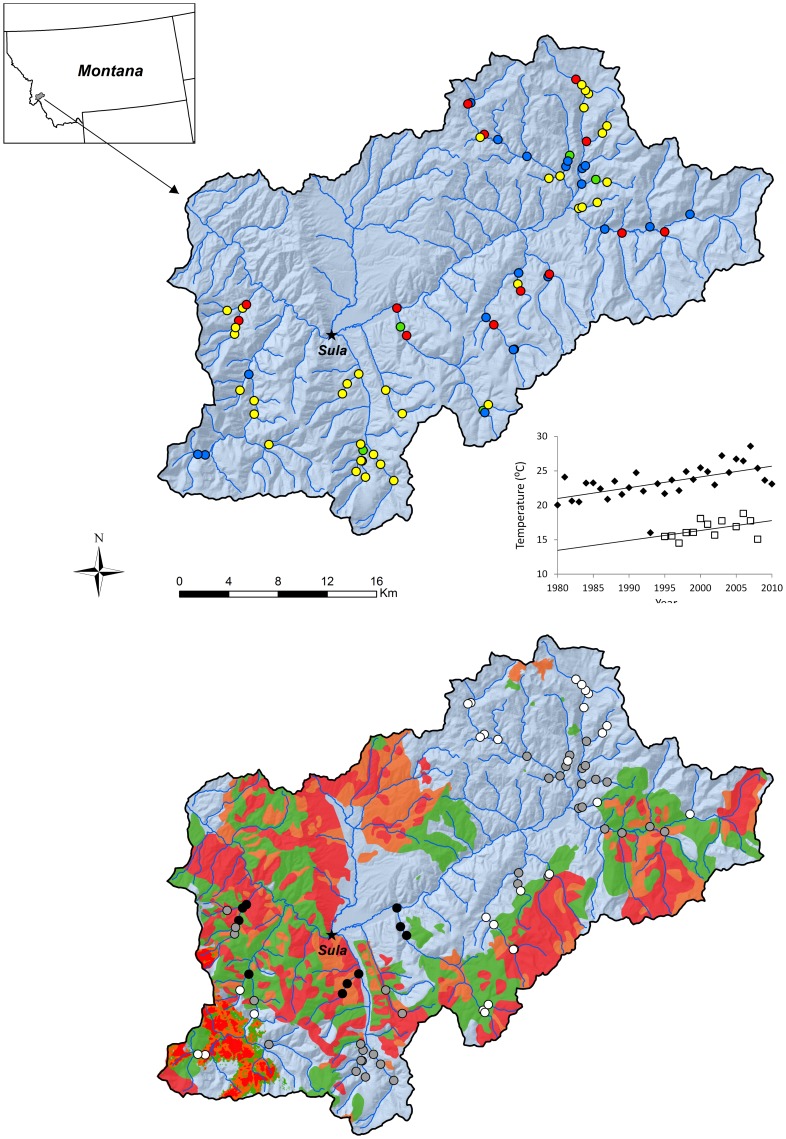
Study area. Sampling locations (500-m sites that were initially visited in 1992–1995 and resampled in 2009–2011) in the East Fork Bitterroot River watershed. Top panel: patterns in occupancy (yellow, not occupied in either period; blue, occupied in both periods; red, occupied in first but not second period; green, occupied in second but not first period). These reflect observed patterns not corrected for probability of detection. Water and air temperature patterns within the East Fork Bitterroot River basin are inset. Black diamond symbols are average daily summertime (July and August air temperature recorded over the study period at the closest weather station at Sula, MT (*y*  =  0.1567*x* – 289.27, *R^2^* = 0.34, *p* = 0.0006). Hollow squares are average daily maximum water temperatures over the summer season (July 15 to September 30) from the East Fork Bitterroot River main stem 28.6 km upstream of the confluence with the West Fork Bitterroot River (*y*  =  0.1441*x* – 271.96, *r^2^* = 0.22, *p* = 0.10). Bottom panel: sampling locations shaded to indicate estimates of abandonment probability (white: 0.21–0.32, grey: 0.32–0.47, and black: 0.47–0.62). Burn severity for fires in the watershed is indicated by low severity in green, moderate severity in orange, and high severity in red. Grey is outside of the fire perimeters.

The East Fork Bitterroot River basin is a 1,055-km^2^ watershed in west-central Montana, U.S.A. (Figure 1). The basin is mainly a forested landscape with lower elevations dominated by ponderosa pine (*Pinus ponderosa*) and Douglas-fir (*Pseudotsuga menziesii*) and higher elevations by lodgepole pine (*Pinus contorta* var. *latifolia*), subalpine fir *(Abies lasiocarpa)*, and Engelmann spruce (*Picea engelmannii*). The watershed is a temperate, snowmelt-dominated system with a range of elevations from 1,220 to 2,887 m. In 2000, wildfires burned 52.0% (29.2% at moderate to high severity) of the basin and 3.8% (2.5% at moderate to high severity) in 2007. Maximum summer stream temperatures in reaches where moderate- to high-severity fires burned in riparian stands remain elevated 1.4 to 2.2°C above those from reaches adjacent to unburned stands [22]. Over a comparable interval (1994–2007) maximum summer stream temperatures at some unburned sites also increased 1.9–2.6°C [22], which is higher than the July/August 0.24°C/decade increase described for the Greater Yellowstone area [26] and 0.22°C/decade increase across the U.S. Northwest [15]. Average daily maximum summer water temperature have been increasing in recent years in the main-stem East Fork Bitterroot River, as have summer air temperatures at the weather station nearest our study area (Sula, MT; Figure 1).

The East Fork Bitterroot River is a core conservation area for bull trout [27]. This watershed consists primarily of public land administered by the U.S. Forest Service (USFS) and has no known barriers to fish movement within our study area [27]. Bull trout in this basin exhibit partial migration, with resident and migratory individuals in most spawning tributaries. Resident individuals spend their entire lives within their natal stream or tributary, moving only short distances (e.g., <2 km), whereas migratory individuals spawn in headwater tributaries but migrate to the river to forage [28], [29]. Other native fish in the basin include westslope cutthroat trout *Oncorhynchus clarkii lewisi*, slimy sculpin (*Cottus cognatus*), longnose dace (*Rhinichthys cataractae*), mountain whitefish (*Prosopium williamsoni*), and longnose suckers (*Catostomus catostomus*). Non-native brown trout (*Salmo trutta*), brook trout, and rainbow trout (*O. mykiss*) are present throughout the main stem and in several tributaries in the basin.

A number of factors influence bull trout habitat occupancy in its U.S. range. As noted earlier, site occupancy of juvenile or resident bull trout is strongly correlated with maximum temperatures [21], [30]. However large individuals with migratory life histories are not restricted to cold thermal environments and can move through much warmer waters (e.g., 21°C 7-day average daily maximum temperature; [31]) before reaching spawning areas. Bull trout are also associated with relatively large patches of connected, complex habitat [17], [32]. In the Bitterroot River basin, the probability of bull trout presence in stream reaches was positively correlated with large wood, stream width, and relative abundance of main-stem bull trout at a tributary mouth, and negatively correlated with stream gradient and the presence of brook trout [33]. Bull trout in this basin tend to occupy streams to their headwaters (until stream width < 2 m; [33]), thus increases in occupancy are only likely at downstream locations or in previously unoccupied streams.

### Data collection

First- through 4^th^-order streams were sampled between 1992 and 1995 to determine bull trout occupancy patterns in the Bitterroot River basin [33]. In this sampling, three 500-m study sites were equally spaced over the estimated length of suitable habitat in each tributary. Between 2009 and 2011, we revisited 74 sites on streams sampled previously [33] within the connected portions of the East Fork Bitterroot River basin to examine whether bull trout occupancy had changed. We relocated the sites and replicated the sampling methods of the earlier study. Fish were collected with a single-pass survey using a backpack electrofishing unit during the summer low-flow period. Care was taken to electroshock slowly and inclusively through all areas of cover. Our resampling was confined to 1^st^- through 3^rd^-order streams that were small enough to effectively sample with these techniques. As in the previous study, we divided each site into five sequential 100-m sections for sampling. All fish were identified to species, counted, and measured (total length). As in the earlier study, we avoided basing bull trout occupancy on the ephemeral presence of a large, migratory adult; presence in a section was defined by the capture of ≥ 2 bull trout, at least one of which was less than 250 mm (and thus likely to be a juvenile or small resident adult of local origin) [21]. All sampling was performed in accordance with guidelines specified under scientific collection permits issued to Lisa Eby by Montana Fish, Wildlife and Parks and under the protocols approved in the University of Montana Institutional Animal Care and Use Committee AUP 031-09.

We used field measures and GIS spatial data layers to assess covariates potentially related to bull trout occupancy. We counted large wood in the first 100-m section of every 500-m site. We estimated bankfull width (m) for the section by measuring it at three representative locations. Elevation (m) was noted in the field from the GPS unit (Garmin 60CSx) and validated from the 30-m cell size National Elevation Dataset [34]. Gradient at each site was derived from this dataset using TauDEM software [35]. We used estimates of bull trout abundance in the main-stem East Fork Bitterroot River (http://fwp.mt.gov/fishing/mFish/newSearch.html; accessed 8/2/13) to assess the proximity of sites to main-stem locations where adults were commonly captured. We categorized sites into three groups: (0) bull trout common in the main-stem East Fork Bitterroot River at the tributary mouth; (1) bull trout common in the main-stem within 2.5 km of the tributary mouth; and (2) bull trout common in the main-stem > 2.5 km from the tributary mouth. Because we did not have a single year with temperature data at every site and annual variation in temperature is large, we used a locally calibrated stream temperature model that allowed standardized representation of relative temperatures among sites (Text S1).

We obtained fire severity GIS layers from the Bitterroot National Forest (Hamilton, MT). Burn severity is used to describe the amount of fire-related change including overstory vegetation mortality, soil heating, and fuel consumption [36], [37]. Our sites within moderate- and high-severity riparian burns had the majority of the riparian area (and watershed) burned, thus fire severity and proportion of site burned were positively related. We grouped burn severity into two categories and at each site we indicated whether the riparian area experienced no-to-low severity burns or medium-to-high severity burns.

### Data analyses

We used program PRESENCE 4.1 [38] to estimate detection probability, occupancy, and abandonment and colonization probabilities. We constructed a survey history based on the five, 100-m sections for each site across the 1992–1995 and 2009–2011 surveys to estimate these parameters. These separate survey intervals were regarded as seasons. Estimates for probability of detection were modeled as a function of standardized values for season, large wood, fire, width, and gradient. Because large wood and the occurrence of medium- to high-severity fire at the site change over time, we treated these as survey-specific covariates. Given that both survey events had imperfect detectability and different crews (but the same field protocol), we compared detection probability between the earlier and more recent surveys. In addition, we explored all possible combinations of covariates in competing models to examine which covariates best described the probability of detection across sites (based on maximum likelihood estimators and Akaike Information Criterion (AIC) [39].

We used a multi-season model to estimate site abandonment and colonization probabilities of bull trout. The model uses initial occupancy estimates for the first sampling period and derives estimates for the abandonment and colonization probabilities that determine whether a species occurs at a site during the second sampling period [40]. To examine changes in occupancy in our data set, we first determined whether estimates of bull trout abandonment or colonization probabilities were significantly different from zero and from one another. Any changes were regarded as apparent because occupancy was only estimated twice, not at repeated intervals. Finally, we tested whether large wood, gradient, width, brook trout presence, proximity to where bull trout were common in the main-stem, occurrence of medium- or high-severity fire, or relative temperature co-varied with these probabilities. In addition, we examined how elevation, an occasional surrogate for water temperature [21], degree of connectivity, channel gradient, and human land use, individually co-varied with colonization and abandonment probabilities. Models were fit using maximum likelihood estimators and ranked based on AIC scores. We considered all models within 2 AIC units of the top model, but disregarded uninformative parameters i.e., covariates for which approximate 85% confidence intervals overlapped zero [41]. We also performed stepwise variable removal, based on the minimum absolute value of β/SE, stopping when variable exclusion led to a decrease in AIC score for the model [41].

## Results

Presence of bull trout in previously occupied sites declined (from 33 sites in 24 streams to 20 sites in 22 streams), and absence from previously unoccupied sites decreased (from 41 sites in 26 streams to 36 sites in 26 streams). Brook trout occupancy declined between the earlier (12 sites in 6 streams) and later (7 sites in 5 streams) surveys. In addition, thirty-one of the 74 sites had adjacent riparian burns during the period between surveys, 12 of which were from medium- to high-severity fires.

Significant correlations among covariates used to explain probability of detection were weak or absent (Table S1), so all were considered in models of detection probability. There were three top models (within 2 AIC units of each other; Table 1). Our naïve (no covariates) probability of detection estimate was 0.54 (SE, 0.03). Naïve detection probabilities did not differ between seasons (season 1: 0.54, SE 0.04; season 2: 0.54, SE 0.06) and season did not increase the model AIC score. Therefore, the top model for estimating probability of detection included only large wood and width as covariates. Based on this model, site-specific probability of detection varied from 0.09 to 0.99 (Table S2).

**Table 1:** Models within two Akaike Information Criterion (AIC) units of the top model for estimating probability of detection of bull trout with probability of occupancy, colonization, and abandonment held constant.

Model^a^^,^ b	AIC	DeltaAIC	AIC Weight	Model likelihood	Parameters	-2Loglikelihood
p(W _4.22_,LW_4.47_)^c^	479.22	0.00	0.5467	1.0000	6	467.22
p(S_0.55_ W _4.22_,LW_4.47_)	480.88	1.66	0.2384	0.436	7	468.04
p(W _4.22_,LW_4.47_,G _0.35_)	481.09	1.87	0.2146	0.393	7	467.09

a Variables subscripted with β/SE absolute values; variables with values < 1.4 are regarded as uninformative (Arnold 2010).

b Abbreviations: W, width; LW, large wood; S, season; G, gradient.

c Only this model lacks uninformative variables.

**Figure 2:**
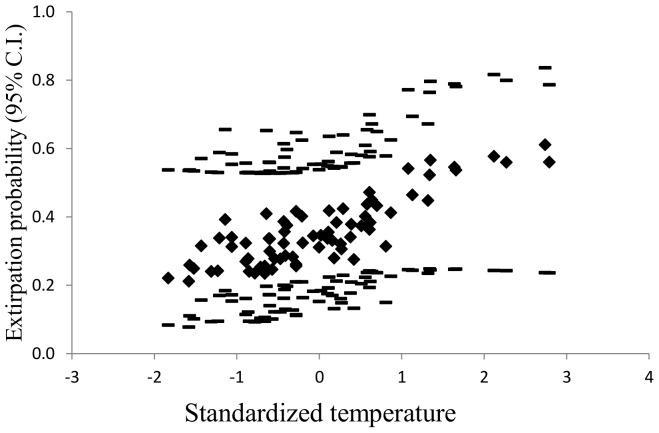
Effect of temperature on abandonment probabilities. Model-averaged abandonment probabilities (filled diamonds) from the top three informative models (Table 2) with their upper and lower 95% confidence intervals (dashes) versus standardized relative temperature across sites in the East Fork Bitterroot River basin.

**Figure 3:**
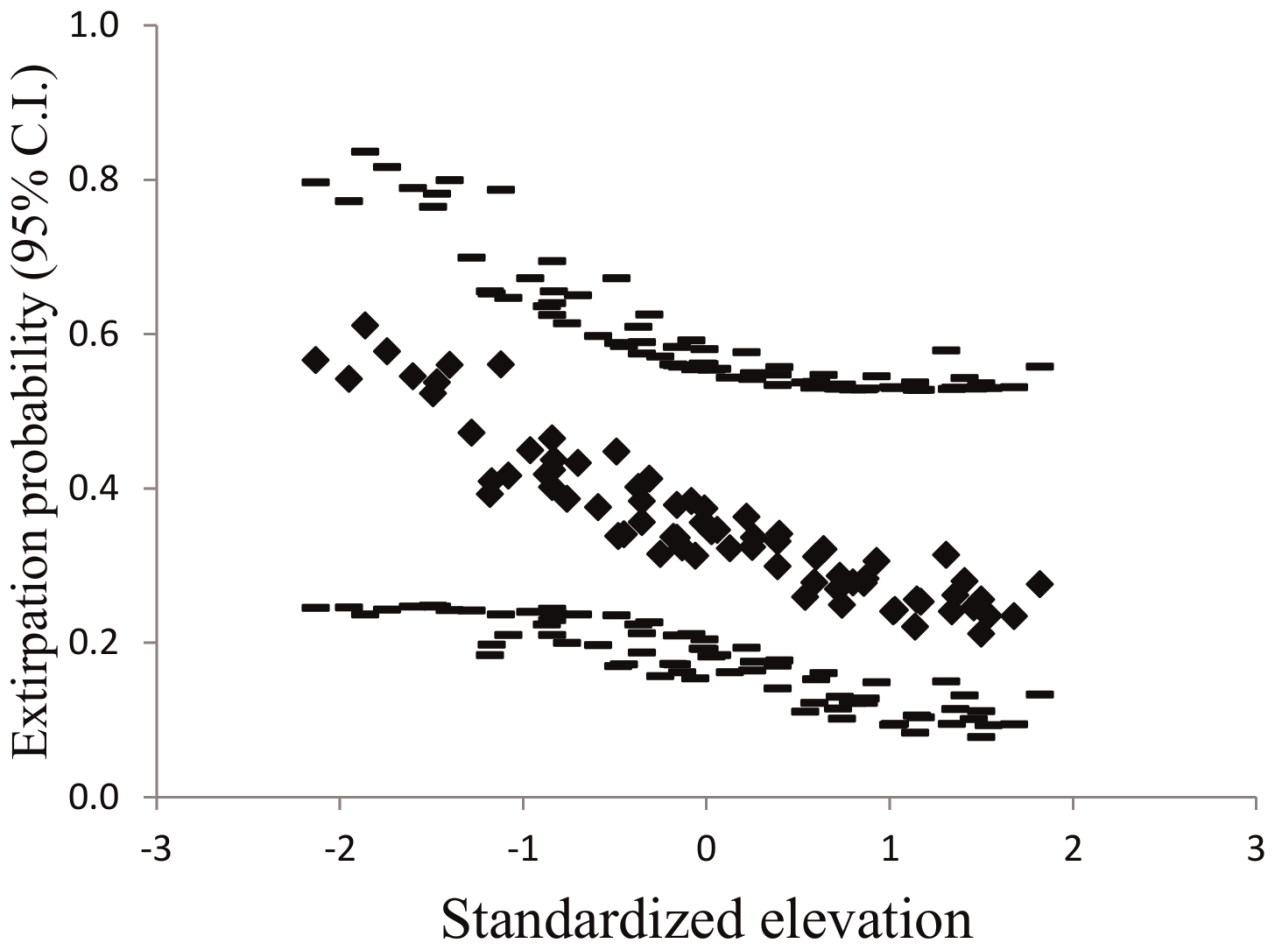
Effect of elevation on abandonment probabilities. Model-averaged abandonment probabilities (filled diamonds) from the top three informative models (Table 2) with their upper and lower 95% confidence intervals (dashes) versus standardized elevation.

The overall (no site or survey covariates) abandonment probability (0.36, SE 0.07) was almost 3-fold greater than that for colonization (0.13, SE 0.07). Most covariates in models for estimating colonization or abandonment probabilities were uninformative because of small effect sizes or large standard errors. The three top models with informative covariates for abandonment probabilities included either no covariate or the single covariates of elevation or temperature (Table 2). We model-averaged the top three models with informative parameters. Estimated abandonment probabilities increased approximately three-fold from cooler to warmer sites (Figure 2) and high- to low-elevation sites (Figure 3). Elevation and temperature were negatively correlated (−0.59). No informative covariates were retained in the top model for estimating colonization.

**Table 2:** Models within two AIC units of the top model for using changes in occupancy (ψ) to estimate colonization (γ) and abandonment (ε) probabilities of bull trout.

Model^a^^,^ b ^,^ c	AIC	DeltaAIC	AIC weight	Model likelihood	Parameters	-2Loglikelihood
ψ, γ,ε(E _1.59_)^d^	478.38	0	0.10	1	7	464.38
ψ,γ,ε(B _0.5_)	478.62	0.24	0.088	0.88	7	464.62
ψ,γ,ε(T _1.43_)^d^	478.94	0.56	0.075	0.76	7	464.94
ψ,γ,ε^d^	479.22	0.84	0.065	0.66	6	467.22
ψ,γ,ε(B _0.47_, D_0.73_)	480.11	1.73	0.042	0.42	8	464.11
ψ,γ,ε(B _0.46_, T _0.72_)	480.12	1.74	0.042	0.42	8	464.12
ψ,γ,ε(B _0.66_, F _0.73_)	480.12	1.74	0.042	0.42	8	464.12
ψ,γ,ε(D_1.04_)	480.17	1.79	0.040	0.41	7	466.17
ψ,γ,ε(B_0.24_,G_0.62_)	480.24	1.86	0.039	0.40	8	464.24
ψ,γ,ε(T_1.43_, LW_0.68_)	480.45	2.0	0.035	0.36	8	464.45

a All models contained the probability of detection function p(W, LW).

b Variables subscripted with β/SE absolute values.

c Abbreviations: E, elevation; T, temperature; LW, large wood; W, stream width; G, gradient; B, brook trout presence at site; D, distance to where bull trout are common in the main-stem East Fork Bitterroot River; F, occurrence of moderate- to high-severity fire.

d Models without uninformative variables. Model-averaged parameter estimates for the untransformed coefficients: E, −0.73 (0.46 SE); T, 0.63 (0.44 SE).

## Discussion

By revisiting historically sampled sites within a river network that encompasses a broad temperature and elevation gradient, we demonstrated that site abandonment probabilities of bull trout were highest at warmer, low-elevation sites over the last two decades. This coincided with increases in summer stream temperatures in the East Fork Bitterroot River basin. Neither colonization nor abandonment probabilities were related to variables reflecting habitat, biotic interactions, or recent disturbance, and probabilities of abandonment were three-fold greater than those of colonization. Collectively, these results represent the first empirical evidence supporting predicted declines in the distribution of bull trout as a consequence of climate change [17], [26], [42]. We acknowledge that the observed relation between climate-related warming and reduction in bull trout occupancy is correlative and that other covariates we did not consider may have influenced this outcome, but the effects of temperature on bull trout distributions are consistent across its historical range [17]. A complete mechanistic understanding of the effects of warming temperatures on bull trout has not been realized, but the restricted scope for growth of juvenile life history stages at warm temperatures is clear [4], [43]. Given that much of the historical range of bull trout is undergoing relatively rapid warming as a consequence of climate change [14], [26], testing the generality of our observations will be straightforward if comparable long-term data sets become available.

Only those covariates most closely related to the decreased thermal suitability of bull trout habitat—water temperature and elevation—appeared in the top models for estimating abandonment probabilities. This may seem surprising, given that occupancy models for bull trout have included an array of habitat and biotic variables [18], [33]. We did not, however, model where bull trout are currently found, but examined what influenced recent changes in that distribution. Consequently, it might be expected that elevation (represented in a linear function across a broad range) would not explain the distribution of bull trout in the Bitterroot River basin [33], but was a top predictor of locations abandoned by bull trout. We attribute most of the explanatory power of elevation in our model to its relation to water temperature, but acknowledge that it can, in part, represent effects of other variables, such as the presence of nonnative species, the effects of fire, or proximity to population strongholds. In this study, however, none of these were informative contributors to models of changes in bull trout occupancy. At the sites we examined, brook trout occupancy was low and appeared to decline during the study. Elsewhere, replacement of bull trout by brook trout appears to be associated with particular valley morphologies [18], [25], that may not be prevalent in the study area. We regard it as unlikely that warming temperatures also reduced brook trout occupancy [24] because this species prefers warmer temperatures than does bull trout [4]. More plausible is that brook trout were declining in response to fire effects [44] but this species was too poorly represented in the data to evaluate this trend.

Stand-replacing fires tend to cause warmer stream temperatures [22], [45], thus it might be expected that reductions in bull trout occupancy would be associated with fire directly, or indirectly via fire's relation to elevation. Although low-elevation sites adjacent to burned areas were some of our warmest sites, there was no significant correlation (*r* = 0.45) between relative temperature and fire because wildfires burned across the entire watershed. Moreover, changes in water temperature may be ameliorated by other fire-related changes in habitat, such as increased autochthonous productivity, macroinvertebrate community shifts, or channel alteration [46], and previous observations of bull trout in the study area did not reveal population declines following fire [44]. Nevertheless, because stand-replacing fire in riparian zones leads to chronic increases in summer stream temperatures it has the potential to contribute to local, site-specific changes in occupancy by bull trout. In addition, anticipated increases in fire extent or frequency attributable to climate change [23], [47] may lead to more profound shifts, or outright extirpations, of populations across the landscape, where populations are isolated or landscapes are prone to large, post-fire debris flows [21].

Connectivity has long been thought to influence the persistence of salmonid populations because these fishes can be highly mobile and frequently form metapopulations [48]. In our analyses, rank distance to the main-stem river sections where bull trout are common did not account for the probability of abandonment or colonization at tributary sites. It may have been that the distances involved (a few tens of kilometers at most) do not represent meaningful levels of isolation for bull trout, migratory forms of which often traverse much longer distances [31], [49]. Genetic evidence indicates that despite declines in the abundance of migratory bull trout in the East Fork Bitterroot River basin over the last few decades, dispersal by bull trout among tributaries and between tributaries and the main-stem East Fork Bitterroot River remains common [50]. If climate change or anthropogenic habitat alteration increases the energetic and demographic costs of migration beyond some threshold, the influence of connectivity may become more evident [51].

An ongoing paradox is that demographic shifts among freshwater species have been difficult to detect despite that these taxa may be among the most sensitive to climate change [11]. Although a few studies have suggested that declines in freshwater fish abundance could be related to climate change [52], [53], [54], beyond the present study only one other [55] has reported changes in their distributions. This could be partly attributable to a paucity of adequately georeferenced historical data sets, particularly those that permit detectability estimation. Nevertheless, the enormity of current and historical fish monitoring efforts by state, tribal, and federal agencies throughout North America suggests that many such data sets exist. For areas lacking historical temperature data, recent advances in modeling dendritic ecological networks [56] can facilitate accurate hindcasting and prediction of stream temperatures for basins well represented by recent temperature records [21].

Other obstacles to detecting the effects of climate change on cold-water fishes reflect their habitat and biology. Although rising water temperature appears to be a consistent trend in many portions of the historical range of bull trout and other western North American salmonids [15], [26], uncertainty about the response of particular watersheds [57] or certain species [55] remains high. Fish abundance is exceptionally temporally variable and sometimes requires decades of sampling before statistically significant trends emerge [58], [59]. In addition, such trends may be superimposed on long-term variation in abundance dictated by climate cycles such as the Pacific Decadal Oscillation [60]. Nevertheless, correlations between bull trout abundance and broad-scale climate cycles (or this species' abundance and that of other salmonids) are weak [61], [62], [63]. Analyses that rely on occupancy, rather than abundance, may be less vulnerable to temporal fluctuations.

In summary, we found that in this core, connected conservation area for bull trout, patterns in site abandonment were consistent with the predicted effects of stream temperature warming. Extending the current time series of observation of bull trout in the East Fork Bitterroot River basin, as well as similar studies in other basins, is essential to evaluating the generality of this trend. Monitoring designs that focus on stenothermic species and on locations most likely to undergo rapid change offer the greatest power for detecting responses of aquatic species to climate change [11]. For bull trout, these include low-elevation reaches that are warming rapidly and high-elevation reaches undergoing flow reductions from declining snowpacks [64]. In the absence of such targeted designs, however, revisiting historically sampled sites across a range of elevations within a stream network to examine changes in occupancy constitutes a practical alternative [10], [65], [66].

## Supporting Information

Table S1 **Correlation coefficients.** Pearson correlation coefficients for standardized variables in analyses including elevation (E), large wood (LW), bank-full width (W), gradient at site (G), relative temperature (T), the presence of medium to high severity burns at the site (F), the presence of brook trout (B), and the distance from the tributary confluence to where bull trout are common in the main-stem (D). An asterisk indicates a significant correlation (*P*≤0.05).
(DOCX)

Table S2 **Detection probabilities.** Detection probabilities associated with each site and each survey considering large wood during each survey and stream width at each site. Probability of detection without covariates was 0.54 (SE, 0.03).
(DOCX)

Text S1 **Stream temperature model predictions.**
(DOCX)
