# Supplementary material for: Evidence of Climate-Induced Range Contractions in Bull Trout Salvelinus confluentus in a Rocky Mountain Watershed, U.S.A
Source: PLoS One. 2014 Jun 4;9(6):e98812. doi: 10.1371/journal.pone.0098812 (PMC4045800; doi:10.1371/journal.pone.0098812)
Supplement: Table S1 — Correlation coefficients. Pearson correlation coefficients for standardized variables in analyses including elevation (E), large wood (LW), bank-full width (W), gradient at site (G), relative temperature (T), the presence of medium to high severity burns at the site (F), the presence of brook trout (B), and the distance from the tributary confluence to where bull trout are common in the main-stem (D). An asterisk indicates a significant correlation (P≤0.05). (DOCX) [file pone.0098812.s001.docx]

**Table S1. Correlation coefficients.**

Pearson correlation coefficients for standardized variables in analyses including elevation (E), large wood (LW), bank-full width (W), gradient at site (G), relative temperature (T), the presence of medium to high severity burns at the site (F), the presence of brook trout (B), and the distance from the tributary confluence to where bull trout are common in the main-stem (D). An asterisk indicates a significant correlation (*P* ≤ 0.05).

|  | E | LW | W | G | T | F | B | D |
| --- | --- | --- | --- | --- | --- | --- | --- | --- |
| E | 1.00 |  |  |  |  |  |  |  |
| LW | 0.14 | 1.00 |  |  |  |  |  |  |
| W | -0.03 | 0.34 | 1.00 |  |  |  |  |  |
| G | 0.13 | 0.08 | -0.12 | 1.00 |  |  |  |  |
| T | -0.59^*^ | -0.02 | 0.16 | -0.19 | 1.00 |  |  |  |
| F | -0.25 | 0.13 | 0.03 | 0.05 | 0.45 | 1.00 |  |  |
| B | -0.45 | -0.10 | 0.27 | -0.17 | 0.56^*^ | 0.11 | 1.00 |  |
| D | -0.46 | -0.24 | 0.06 | 0.16 | 0.31 | 0.17 | 0.16 | 1.00 |
