# Supplementary material for: Evidence of Climate-Induced Range Contractions in Bull Trout Salvelinus confluentus in a Rocky Mountain Watershed, U.S.A
Source: PLoS One. 2014 Jun 4;9(6):e98812. doi: 10.1371/journal.pone.0098812 (PMC4045800; doi:10.1371/journal.pone.0098812)
Supplement: Table S2 — Detection probabilities. Detection probabilities associated with each site and each survey considering large wood during each survey and stream width at each site. Probability of detection without covariates was 0.54 (SE, 0.03). (DOCX) [file pone.0098812.s002.docx]

**Table S2. Detection probabilities.**

Detection probabilities associated with each site and each survey considering large wood during each survey and stream width at each site. Probability of detection without covariates was 0.54 (SE, 0.03).

| Survey and site | Probability of detection | Lower 95% CI | Upper 95% CI | Survey and site | Probability of detection | Lower 95% CI | Upper 95% CI |
| --- | --- | --- | --- | --- | --- | --- | --- |
| 1–1 | 0.457 | 0.357 | 0.561 | 2–1 | 0.327 | 0.240 | 0.427 |
| 1–2 | 0.663 | 0.539 | 0.769 | 2–2 | 0.688 | 0.557 | 0.794 |
| 1–3 | 0.759 | 0.651 | 0.842 | 2–3 | 0.595 | 0.517 | 0.669 |
| 1–4 | 0.810 | 0.711 | 0.881 | 2–4 | 0.990 | 0.942 | 0.998 |
| 1–5 | 0.636 | 0.552 | 0.713 | 2–5 | 0.762 | 0.665 | 0.837 |
| 1–6 | 0.501 | 0.398 | 0.604 | 2–6 | 0.340 | 0.254 | 0.437 |
| 1–7 | 0.529 | 0.371 | 0.681 | 2–7 | 0.940 | 0.857 | 0.977 |
| 1–8 | 0.471 | 0.386 | 0.558 | 2–8 | 0.408 | 0.326 | 0.495 |
| 1–9 | 0.341 | 0.258 | 0.436 | 2–9 | 0.343 | 0.260 | 0.437 |
| 1–10 | 0.440 | 0.355 | 0.528 | 2–10 | 0.400 | 0.318 | 0.489 |
| 1–11 | 0.508 | 0.415 | 0.601 | 2–11 | 0.796 | 0.639 | 0.896 |
| 1–12 | 0.676 | 0.586 | 0.755 | 2–12 | 0.498 | 0.420 | 0.577 |
| 1–13 | 0.490 | 0.414 | 0.566 | 2–13 | 0.413 | 0.332 | 0.500 |
| 1–14 | 0.839 | 0.709 | 0.918 | 2–14 | 0.845 | 0.714 | 0.922 |
| 1–15 | 0.136 | 0.069 | 0.251 | 2–15 | 0.148 | 0.077 | 0.266 |
| 1–16 | 0.739 | 0.646 | 0.815 | 2–16 | 0.841 | 0.736 | 0.909 |
| 1–17 | 0.943 | 0.858 | 0.979 | 2–17 | 0.857 | 0.714 | 0.935 |
| 1–18 | 0.394 | 0.269 | 0.534 | 2–18 | 0.676 | 0.586 | 0.756 |
| 1–19 | 0.283 | 0.196 | 0.389 | 2–19 | 0.368 | 0.286 | 0.458 |
| 1–20 | 0.210 | 0.130 | 0.320 | 2–20 | 0.384 | 0.283 | 0.495 |
| 1–21 | 0.908 | 0.784 | 0.964 | 2–21 | 0.715 | 0.612 | 0.800 |
| 1–22 | 0.532 | 0.457 | 0.606 | 2–22 | 0.570 | 0.492 | 0.644 |
| 1–23 | 0.503 | 0.427 | 0.578 | 2–23 | 0.450 | 0.371 | 0.532 |
| 1–24 | 0.497 | 0.409 | 0.585 | 2–24 | 0.575 | 0.472 | 0.672 |
| 1–25 | 0.198 | 0.118 | 0.313 | 2–25 | 0.212 | 0.129 | 0.329 |
| 1–26 | 0.921 | 0.821 | 0.967 | 2–26 | 0.888 | 0.784 | 0.945 |
| 1–27 | 0.412 | 0.278 | 0.561 | 2–27 | 0.580 | 0.471 | 0.681 |
| 1–28 | 0.364 | 0.222 | 0.534 | 2–28 | 0.609 | 0.499 | 0.708 |
| 1–29 | 0.806 | 0.602 | 0.919 | 2–29 | 0.885 | 0.740 | 0.954 |
| 1–30 | 0.435 | 0.346 | 0.529 | 2–30 | 0.503 | 0.404 | 0.602 |
| 1–31 | 0.639 | 0.556 | 0.714 | 2–31 | 0.647 | 0.564 | 0.722 |
| 1–32 | 0.370 | 0.289 | 0.460 | 2–32 | 0.491 | 0.409 | 0.572 |
| 1–33 | 0.556 | 0.459 | 0.648 | 2–33 | 0.393 | 0.312 | 0.481 |
| 1–34 | 0.581 | 0.481 | 0.675 | 2–34 | 0.370 | 0.288 | 0.460 |
| 1–35 | 0.134 | 0.069 | 0.245 | 2–35 | 0.244 | 0.159 | 0.355 |
| 1–36 | 0.280 | 0.195 | 0.384 | 2–36 | 0.425 | 0.328 | 0.528 |
| 1–37 | 0.302 | 0.207 | 0.417 | 2–37 | 0.256 | 0.169 | 0.369 |
| 1–38 | 0.564 | 0.473 | 0.651 | 2–38 | 0.544 | 0.450 | 0.634 |
| 1–39 | 0.612 | 0.532 | 0.686 | 2–39 | 0.556 | 0.479 | 0.630 |
| 1–40 | 0.486 | 0.406 | 0.566 | 2–40 | 0.424 | 0.336 | 0.517 |
| 1–41 | 0.751 | 0.656 | 0.826 | 2–41 | 0.577 | 0.476 | 0.672 |
| 1–42 | 0.096 | 0.043 | 0.199 | 2–42 | 0.158 | 0.086 | 0.272 |
| 1–43 | 0.172 | 0.099 | 0.283 | 2–43 | 0.268 | 0.181 | 0.377 |
| 1–44 | 0.189 | 0.112 | 0.300 | 2–44 | 0.235 | 0.152 | 0.347 |
| 1–45 | 0.094 | 0.042 | 0.197 | 2–45 | 0.141 | 0.074 | 0.253 |
| 1–46 | 0.542 | 0.462 | 0.620 | 2–46 | 0.442 | 0.349 | 0.539 |
| 1–47 | 0.550 | 0.472 | 0.627 | 2–47 | 0.490 | 0.406 | 0.575 |
| 1–48 | 0.524 | 0.409 | 0.637 | 2–48 | 0.304 | 0.218 | 0.406 |
| 1–49 | 0.359 | 0.270 | 0.458 | 2–49 | 0.190 | 0.113 | 0.302 |
| 1–50 | 0.278 | 0.172 | 0.417 | 2–50 | 0.170 | 0.094 | 0.289 |
| 1–51 | 0.597 | 0.494 | 0.693 | 2–51 | 0.446 | 0.318 | 0.581 |
| 1–52 | 0.255 | 0.163 | 0.374 | 2–52 | 0.301 | 0.200 | 0.425 |
| 1–53 | 0.133 | 0.067 | 0.246 | 2–53 | 0.180 | 0.100 | 0.303 |
| 1–54 | 0.298 | 0.209 | 0.406 | 2–54 | 0.260 | 0.175 | 0.369 |
| 1–55 | 0.742 | 0.649 | 0.818 | 2–55 | 0.504 | 0.397 | 0.611 |
| 1–56 | 0.521 | 0.442 | 0.599 | 2–56 | 0.457 | 0.370 | 0.547 |
| 1–57 | 0.306 | 0.181 | 0.468 | 2–57 | 0.133 | 0.066 | 0.250 |
| 1–58 | 0.137 | 0.070 | 0.251 | 2–58 | 0.125 | 0.062 | 0.236 |
| 1–59 | 0.190 | 0.110 | 0.308 | 2–59 | 0.179 | 0.101 | 0.295 |
| 1–60 | 0.263 | 0.167 | 0.388 | 2–60 | 0.254 | 0.160 | 0.378 |
| 1–61 | 0.164 | 0.088 | 0.285 | 2–61 | 0.144 | 0.074 | 0.260 |
| 1–62 | 0.122 | 0.059 | 0.238 | 2–62 | 0.150 | 0.077 | 0.274 |
| 1–63 | 0.237 | 0.154 | 0.345 | 2–63 | 0.218 | 0.138 | 0.327 |
| 1–64 | 0.154 | 0.084 | 0.265 | 2–64 | 0.172 | 0.099 | 0.284 |
| 1–65 | 0.158 | 0.087 | 0.269 | 2–65 | 0.245 | 0.158 | 0.360 |
| 1–66 | 0.582 | 0.496 | 0.664 | 2–66 | 0.383 | 0.301 | 0.473 |
| 1–67 | 0.099 | 0.045 | 0.203 | 2–67 | 0.168 | 0.094 | 0.281 |
| 1–68 | 0.606 | 0.439 | 0.752 | 2–68 | 0.302 | 0.207 | 0.417 |
| 1–69 | 0.312 | 0.220 | 0.421 | 2–69 | 0.373 | 0.273 | 0.486 |
| 1–70 | 0.173 | 0.099 | 0.284 | 2–70 | 0.201 | 0.122 | 0.312 |
| 1–71 | 0.438 | 0.320 | 0.563 | 2–71 | 0.251 | 0.165 | 0.362 |
| 1–72 | 0.440 | 0.333 | 0.553 | 2–72 | 0.247 | 0.164 | 0.355 |
| 1–73 | 0.773 | 0.651 | 0.861 | 2–73 | 0.776 | 0.656 | 0.864 |
| 1–74 | 0.805 | 0.669 | 0.895 | 2–74 | 0.740 | 0.579 | 0.854 |
