# Supplementary material for: Evidence of Climate-Induced Range Contractions in Bull Trout Salvelinus confluentus in a Rocky Mountain Watershed, U.S.A
Source: PLoS One. 2014 Jun 4;9(6):e98812. doi: 10.1371/journal.pone.0098812 (PMC4045800; doi:10.1371/journal.pone.0098812)
Supplement: Text S1 — Stream temperature model predictions. (DOCX) [file pone.0098812.s003.docx]

**Text S1. Stream temperature model predictions**

We developed a stream temperature regression equation (Isaak et al. 2010) and linked it to a landscape simulation model which allowed us to predict relative stream temperatures at fish sampling locations (Holsinger & Keane 2011). The regression equation was developed using data from 116 temperature logger sites collected across a range of burn severities and in unburned sites for the years 2000 to 2009, mainly during the summer period July 15–September 30 (U.S. Forest Service, University of Montana, and Montana Fish, Wildlife and Parks, unpublished data). Predictors for the stream temperature regression equation were derived from a spatially explicit landscape model, FireBGCv2, and from topographic features (Keane et al. 2011). FireBGCv2 predictors were air temperature, stream flow, and radiation at the stream surface and the geomorphic predictors included elevation, channel slope, and watershed contributing area. We calibrated the FireBGCv2 model to the East Fork watershed using spatial inputs based on 2004 satellite imagery such that model output represented conditions (loss of canopy cover and increased solar radiation) after the 2000 fires. The daily mean stream temperature (Y, in °C) equation was:

Y = 11.0 + 0.501(daily average air temperature, °C) – 0.236(streamflow, m^3^ sec^-1^) + 0.0191(stream flow × daily average air temperature) + 0.00160(solar radiation, watts m^-2^) – 0.00536(elevation, m) – 2.98(channel slope, drop over length) + 0.00000000166(contributing area, m^2^)

The regression equation accounted for a large portion of the daily stream temperatures variability (daily mean *R^2^* = 0.78, RMSE = 1.54 °C), and validation comparisons with independent data (August 2010 through August 2011) indicated that predictions compared reasonably well to field observations (*r_s_* = 0.83, RMSE = 3.42 °C).

Our goal for using modeled stream temperature in the analysis was to standardize temperatures so that it was possible to accurately portray relative temperature differences among sites that we revisited to explore changes in bull trout occupancy in warmer versus cooler areas. These model-derived temperature differences reflected both site differences associated with landscape position (air temperature, elevation, and contributing area) and solar radiation at a site after the 2000 wildfires. To differentiate sites that were consistently warmer across the landscape, we calculated residuals for each site by subtracting the average basin-wide predicted temperature from the predicted temperature of each site for each year (from 2000 to 2009). We then summed these temperature residuals across the time period 2000–2009 to ensure that sites that were consistently warmer had greater positive residuals and sites that were consistently cooler had larger negative residuals. We used these summed temperature residuals as a covariate in the analysis.

**Literature Cited**

Holsinger L, Keane RE (2011) Fire and fish dynamics in a changing climate. Fire Manage Today 71: 19–23.

Isaak DJ, Luce CH, Rieman BE, Nagel DE, Peterson EE, et al. (2010) Effects of climate change and wildfire on stream temperatures and salmonid thermal habitat in a mountain river network. Ecol Applicat 20: 1350–1371.

Keane RE, Loehman RA, Holsinger LM (2011) The FireBGCv2 landscape fire and succession model: a research simulation platform for exploring fire and vegetation dynamics. U.S. For Serv Gen Tech Rep RMRS-GTR-255.
